# Words and Deeds

**Published:** 1921-01-29

**Authors:** E. F. Gray

**Affiliations:** 10 Tower Street, Alton, Hants


*To the Editor of* The Hospital.

### "Words and Deeds."

*To the Editor of* The Hospital.

Sir,—While it may be true that "British doctors" generally and specialists especially are behind the times "in matters of science," is there not also some fault to be found with the British journals for having failed to help the doctor keep abreast of the times?

Surely some of those journals ought to have been aware of that "medical controversy waged on the Continent, and settled, twenty years ago," which is alleged only just now to have reached England! Is it not such service as this that the busy doctor expects from his journals?

Whose fault was it that "the discussion of bronchoscopy" was "followed at a five-year interval by a working over of the exhausted ground in British journals"?—

Yours faithfully, E. F. Gray.

10 Tower Street, Alton, Hants, January 15

